# Use of Laser Speckle Contrast Imaging for Successful Fingertip Replantation

**DOI:** 10.1097/GOX.0000000000001924

**Published:** 2018-09-05

**Authors:** Ryo Karakawa, Tomoyuki Yano, Hidehiko Yoshimatsu, Mitsunobu Harima, Koji Kanayama, Takuya Iida, Masayuki Sawaizumi

**Affiliations:** From the * Department of Plastic and Reconstructive Surgery, Cancer Institute Hospital of Japanese Foundation for Cancer Research, Tokyo, Japan; † Department of Plastic and Reconstructive Surgery, Kanto Central Hospital, Tokyo, Japan; ‡ Department of Plastic and Reconstructive Surgery, The University of Tokyo, Tokyo, Japan.

## Abstract

Fingertip replantation is a technical challenge for microsurgeons. For successful fingertip replantation, it is important to monitor the replanted fingertip vascularity for the early detection and revision of vascular compromise. Laser speckle contrast imaging (LSCI) is a camera-based technique that measures the perfusion by illuminating the tissue with a 785-nm-wavelength divergent laser beam. This creates a speckle pattern over the illuminated area. We present a case in which postoperative monitoring of the replanted fingertip microcirculation using LSCI allowed for successful Tamai zone I fingertip replantation. Postoperative monitoring using LSCI has 3 main advantages. First, this method is harmless to the patient and the replanted fingertip. A camera-based technique enables microcirculation monitoring without touching the patient or the replanted fingertip. Second, tissue perfusion is measured in real time and recorded continuously, allowing for the rapid response to the arterial or venous occlusion to be observed. Third, using LSCI, the skin perfusion can be measured quantitatively. Although further clinical investigations will be required to confirm its efficacy, LSCI has the potential to be a useful monitoring device.

Fingertip replantation is a technical challenge for microsurgeons. For successful fingertip replantation, it is important to monitor the replanted fingertip vascularity for the early detection and revision of vascular compromise. However, it has been challenging to find a reliable way to monitor the transferred flap or the replanted fingers with high sensitivity and specificity. Compromised vascularity typically occurs within the first 2 days postoperatively, during which time the surgeons must check the viability of the transferred flap or the replanted fingers.^1,2^

Laser speckle contrast imaging (LSCI) is a camera-based technique that illuminates an area of tissue with divergent 785-nm laser light and analyses the interference pattern of the light that is scattered from the tissue. The current literature reports that LSCI offers sensitive and reproducible measurements of flap microcirculation in a porcine flap model.^3^

We herein report a case in which postoperative monitoring of the replanted fingertip microcirculation using LSCI allowed for successful fingertip replantation.

## CASE REPORT

**Fig. 1.**
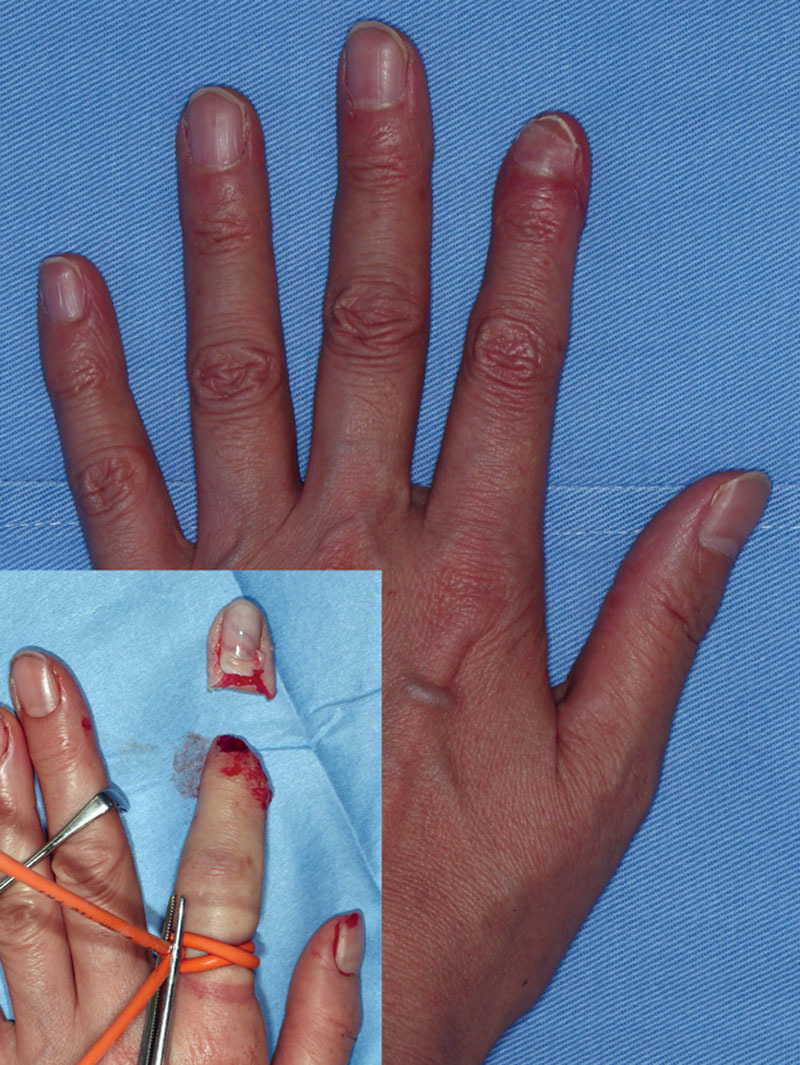
Preoperative view and postoperative view at 5 months. The patient suffered from Tamai zone I amputation of her left second finger.

A 59-year-old woman suffered from Tamai zone I amputation of her left second finger in an accident involving an automatic door at her workplace. Replantation was performed under general anesthesia (Fig. 1). Using a light-emitting diode transilluminator, the recipient veins were visualized preoperatively.^4^ Two arteries, 1 vein, and 1 nerve were repaired. Postoperatively, the fingertip microcirculation was monitored using LSCI immediately after the operation and on the morning after the surgery for 3 days.

**Fig. 2.**
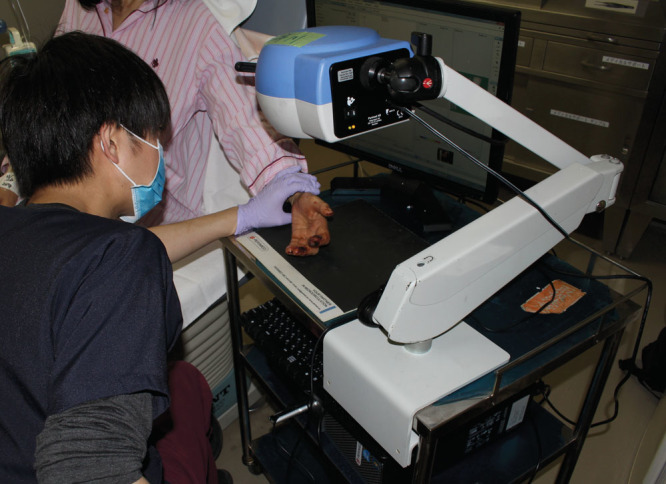
LSCI (PeriCam PSI System, Perimed AB, Sweden).

A Laser Speckle Contrast Imager (PeriCam PSI System; Perimed AB, Sweden) was placed approximately 20 cm above the patient’s hand to measure the perfusion of the fingertip (Fig. 2). LSCI measures the perfusion by illuminating the tissue with a 785-nm-wavelength divergent laser beam. This creates a speckle pattern over the illuminated area. A CMOS camera captures the speckle image, while another captures a conventional color image of the measured area. The principle of this technique has been previously described in detail.^5,6^

The distance between the camera and the patient’s hand was kept at 20 cm, and the image size was set to a 16 × 18-cm area. The frame rate was set to 6 images/s. With each measurement, the perfusion data from 60 consecutive images were averaged, resulting in a total measurement time of 10 s for each image. LSCI images were processed using the system analysis software program (PSIWin; Perimed AB). In each image, 5 circular regions of interest (ROIs) were selected in the left hand: the replanted second finger and the 4 other healthy fingertips. For each image, the average perfusion in each ROI was calculated.

**Fig. 3.**
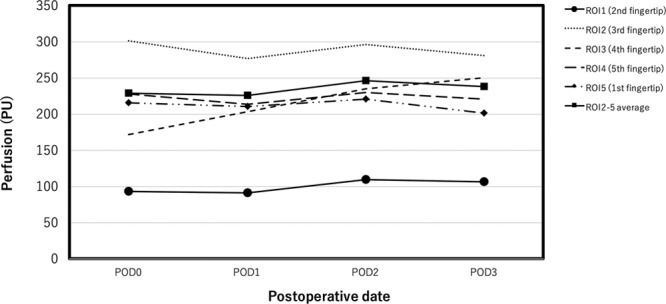
The postoperative change in the skin perfusion of 5 ROI measured by LSCI. PU is proportional to tissue perfusion because it reflects the average velocity and concentration of blood cells. The skin perfusion in the replanted second fingertip was stable postoperatively.

The perfusion in the replanted second fingertip and the average perfusion of the 4 unaffected fingertips immediately after the operation was 93.5 and 229.36 perfusion units, respectively. The postoperative change in the skin perfusion measured by LSCI is presented in Figure 3.

The postoperative course was uneventful, and the perfusion measured with LSCI was stable during the observation period. The replanted fingertip survived successfully (Fig. 1).

## DISCUSSION

In the setting of fingertip replantation, especially in cases without venous anastomosis or with unreliable vessel anastomosis, postoperative monitoring of the replanted fingertip is essential.^7^ Surgeons still rely substantially on clinical controls, such as color changes in the fingertip, changes in the temperature, capillary blink, and the pinprick test. Postoperative monitoring using LSCI has 3 main advantages. First, this method is harmless to the patient and the replanted fingertip. A camera-based technique enables microcirculation monitoring without touching the patient or the replanted fingertip. Second, tissue perfusion is measured in real time and recorded continuously, allowing for the rapid response to the arterial or venous occlusion to be observed.^3^ Third, using LSCI, the skin perfusion can be measured quantitatively. The quantification of the skin perfusion, which used to be evaluated by gross appearance, makes it possible to compare the perfusion in the transferred skin to that in the healthy skin.

**Fig. 4.**
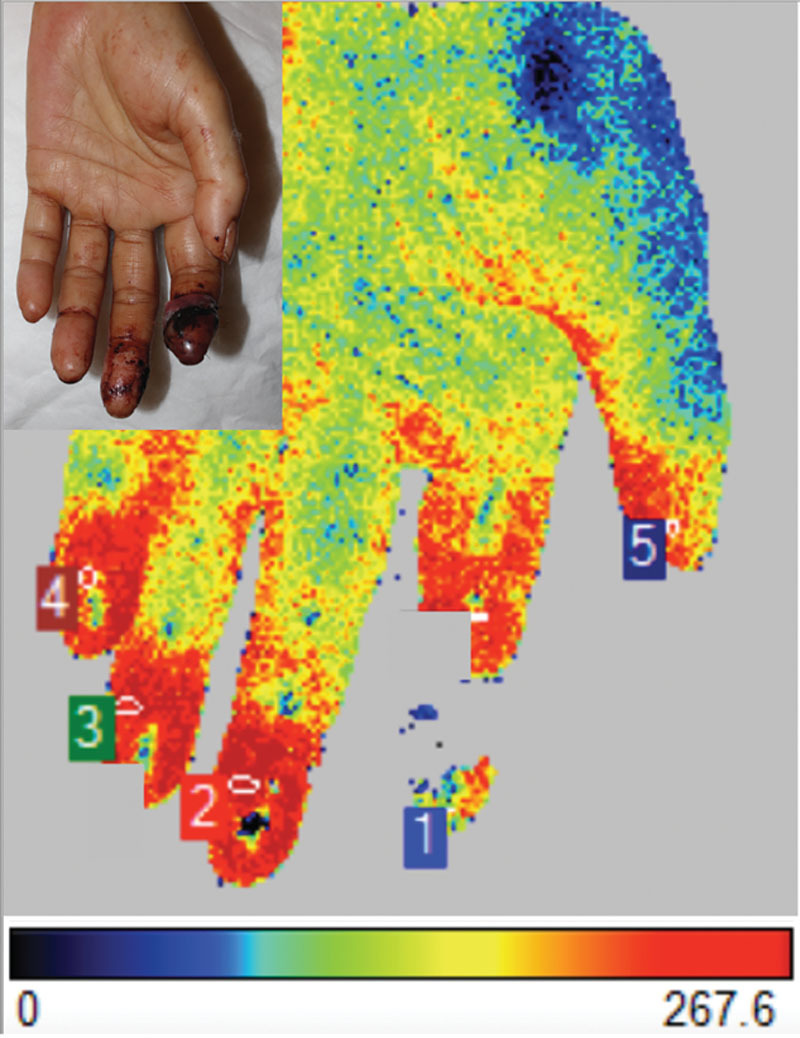
LSCI image of the hand showing typical perfusion image on POD 2. The replanted second fingertip shows less vascularized than the other 4 healthy fingertips. POD, postoperative day.

In the present case, although the skin perfusion of the replanted fingertip was lower than that of the unaffected fingertips, no appreciable decrease in the perfusion of the replanted fingertip was seen during the postoperative period of observation (Fig. 4). A previous study in which LSCI was used to assess the ability to detect venous or arterial occlusion using a porcine flap reported that perfusion decreased to 71% from baseline after venous occlusion and 63% after arterial occlusion.^3^ Therefore, we concluded that there was no problem with the microcirculation during the postoperative period of observation, resulting in the replanted fingertip surviving successfully. As of this moment, the cutoff value of perfusion decrease after arterial or venous occlusion is not clear in clinical cases and further investigation will be needed.

To our knowledge, this is the first report in which successful fingertip replantation was performed with postoperative monitoring of the replanted fingertip microcirculation using LSCI. Although further clinical investigations will be required to confirm its efficacy, LSCI has the potential to be a useful monitoring device.
